# The effect of sodium/glucose cotransporter 2 (SGLT2) inhibition on the urinary proteome

**DOI:** 10.1371/journal.pone.0186910

**Published:** 2017-10-30

**Authors:** David Cherney, Bruce A. Perkins, Yuliya Lytvyn, Hiddo Heerspink, María E. Rodríguez-Ortiz, Harald Mischak

**Affiliations:** 1 Division of Nephrology, University Health Network, University of Toronto, Toronto, Canada; 2 Division of Endocrinology, University Health Network, University of Toronto, Toronto, Canada; 3 Department of Clinical Pharmacology University Medical Center Groningen, Groningen, the Netherlands; 4 Instituto de Investigación Sanitaria Fundación Jiménez Díaz. Fundación Renal Iñigo Álvarez de Toledo. Universidad Autónoma de Madrid. REDinREN, Madrid, Spain; 5 Mosaiques diagnostics GmbH, Hanover, Germany; 6 BHF Glasgow Cardiovascular Research Centre, Institute of Cardiovascular and Medical Sciences, University of Glasgow, Glasgow, United Kingdom; Icahn School of Medicine at Mount Sinai, UNITED STATES

## Abstract

Treatment with empagliflozin, an inhibitor of the sodium/glucose cotransporter 2 (SGLT2), is associated with slower progression of diabetic kidney disease. In this analysis, we explored the hypothesis that empagliflozin may have an impact on urinary peptides associated with chronic kidney disease (CKD). In this *post-hoc*, exploratory analysis, we investigated urine samples obtained from 40 patients with uncomplicated type 1 diabetes (T1D) before and after treatment with empagliflozin for 8 weeks to for significant post-therapy changes in urinary peptides. We further assessed the association of these changes with CKD in an independent cohort, and with a previously established urinary proteomic panel, termed CKD273. 107 individual peptides significantly changed after treatment. The majority of the empagliflozin-induced changes were in the direction of “CKD absent” when compare to patients with CKD and controls. A classifier consisting of these 107 peptides scored significantly different in controls, in comparison to CKD patients. However, empagliflozin did not impact the CKD273 classifier. Our data indicate that empagliflozin induces multiple significant changes in the urinary proteomic markers such as mucin and clusterin. The relationship between empagliflozin-induced proteomic changes and clinical outcomes merits further investigation.

## Introduction

Diabetes-associated vascular diseases, especially chronic kidney disease (CKD) represent a major burden for developed societies [1]. Today, treatment of CKD in diabetes, also referred to as diabetic kidney disease, DKD, is typically initiated when first symptoms are evident: persistent microalbuminuria or decreased glomerular filtration rate (GFR). Standard treatment is reduction of blood pressure by interfering with the rennin/angiotension/aldosterone systems (RAAS). SGLT2 inhibition may represent an additional renoprotective intervention–beyond the use of renin angiotensin aldosterone system blockade–for DKD. [2,3].

Multiple recent reports have demonstrated the potential of proteomic biomarkers in kidney disease, as reviewed in [4]. While individual biomarkers, like e.g. albuminuria, show considerable variability, this variability can be counteracted by employing high-dimensional classifiers. In such an approach, multiple biomarkers are combined in a predefined model, reducing and combining the biomarkers into one variable expressing the probability of the membership in a specific group (e.g. healthy or CKD) [5]. We have previously developed capillary electrophoresis coupled to mass spectrometry (CE-MS) for the routine application in the assessment of clinically relevant samples, mostly urine [6] and dialysis fluid [7]. The results demonstrated that analysis of the urinary proteome enables discrimination between patients with and without CKD [8], as well as prediction of progression of CKD, irrespective of the underlying disease mechanism [9]. The CE-MS technology is especially well suited to assess small proteins and peptides, without the requirement of tryptic digestion [10].

The aim of this *post-hoc*, exploratory analysis was to investigate if treatment with empagliflozin impacts the urinary peptidome, with a special emphasis on urinary peptide biomarkers associated with CKD–specifically the CKD273 biomarker, which is associated with CKD progression [11]. Such an impact could serve as indicative of a potential benefit of empagliflozin prior to onset of CKD, and as potential evidence for the use of empagliflozin in the prevention of DKD onset and progression.

## Materials and methods

### Patients and sample collection

Samples were collected as described in previous work during clamped euglycemia and hyperglycemia in uncomplicated patients with T1D before and after treatment with empagliflozin (25 mg daily) for 8 weeks [12]. All patients consented to the study, which was approved by the University of Toronto University Health Network with approval #11–0213. The available clinical and demographic data were presented previously [12].

### Urinary proteome analysis and peptide identification

The urine samples were prepared and analyzed using a P/ACE MDQ capillary electrophoresis system (Beckman Coulter, Fullerton, USA) on line coupled to a MicroTOF MS (Bruker, Bremen, Germany) described previously in detail [13]. Accuracy, precision, selectivity, sensitivity, reproducibility, and stability of the CE-MS method have been previously described [14]. To normalize for variability in urinary output, a set of 29 internal standard peptides were used for calibration, as previously described [15]. Relative abundance of all peptides in a sample is assessed based on the peak area, and normalized to the 29 standard peptides [15]. This procedure has been applied successfully in multiple previous studies, some based on between 1000 and over 10000 individual samples [16,17]. All detected peptides were deposited, matched, and annotated in a MicrosoftSQL database [9], allowing for further analysis and comparison between groups. Sequencing of target peptides was performed as described [18], using Dionex Ultimate 3000 RSLS nano flow system (Dionex, Camberly UK) and a Beckman CE, coupled to an Orbitrap Velos MS instrument (Thermo Scientific). To assess the distribution of peptides in the context of CKD, previously generated datasets [8] were employed. The clinical and demographic data of this cohort are available from the original publication [8].

### Combining peptides into a classifier

To generate a peptide pattern indicative of the impact of empogliflazin, the support vector machine (SVM)-based MosaCluster software [19] was employed. MosaCluster (version 1.7.0) was developed for discrimination between different patient groups in the high-dimensional parameter space by using SVM learning. SVM generates high dimensional models, which rely on features (biomarkers) displaying statistically significant differences between data from patients with a specific disease to controls or other diseases. Each feature allegorizes one dimension in the n-dimensional parameter space [20]. The two classes (here: prior and after empagliflozin treatment) are separated by an n-1 dimensional separating hyperplane. The position of a dataset (a sample) in the n-dimensional dataspace is defined by the amplitude of the features (the peptides used in the classifier). Classification scores provided by this software give a numerical value quantifying the Euclidean distance of the dataset to the maximal margin of the separation hyperplane among cases and controls in multidimensional space, as defined base on the data in the training cohort. A more detailed description has been published recently [14].

### Statistical analysis

After testing for normal distribution, continuous data were compared by Wilcoxon rank-sum test, as this test has proven to be of superior statistical power in proteomics datasets [5]. A p-value of <0.05 was considered to be statistically significant. In order to control for the false discovery rate, the p-values were adjusted by the Benjamini and Hochberg method [21].

## Results

**Fig 1:**
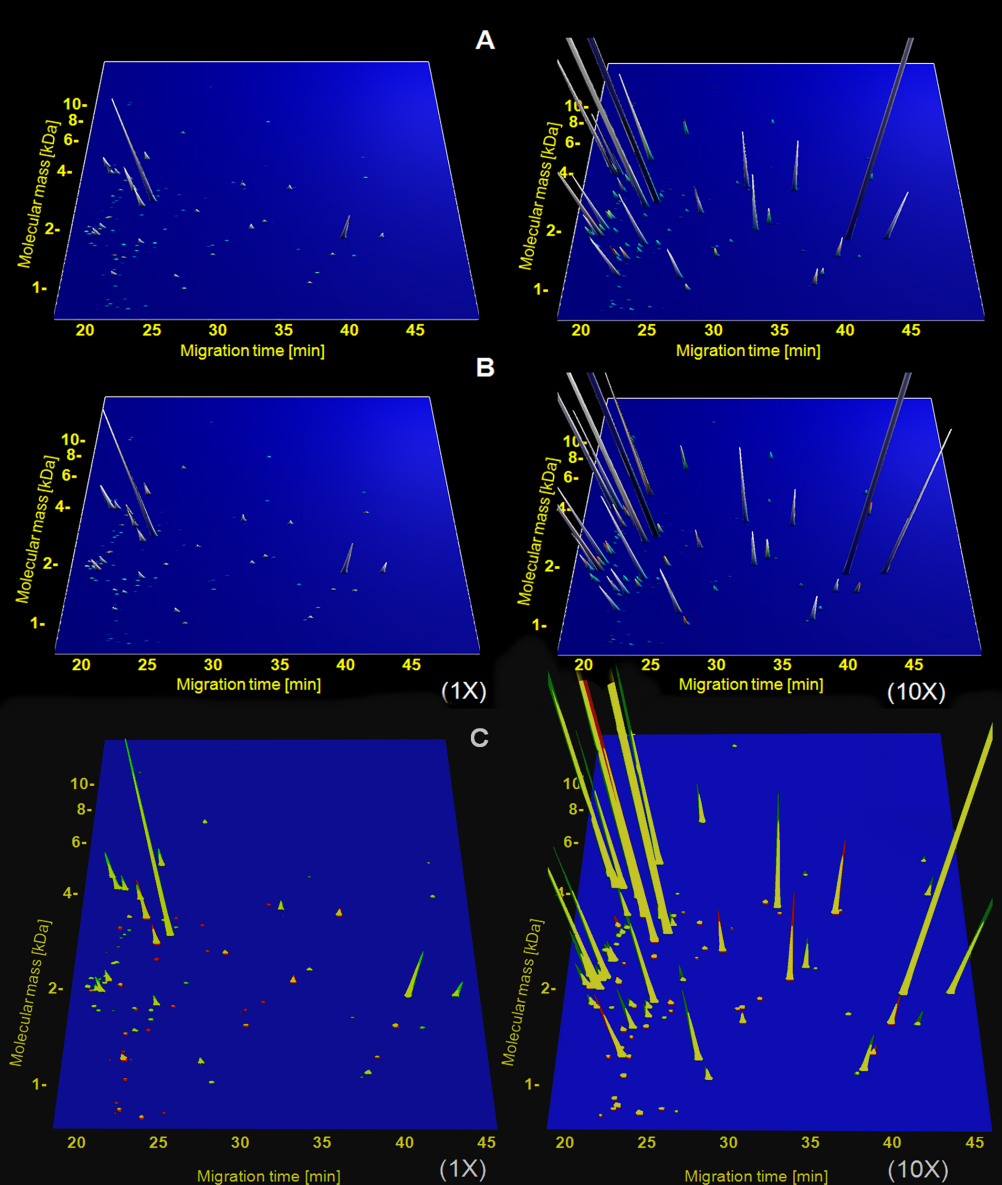
Distribution of the 107 peptides found associated with empagliflozin treatment in the cohort investigated. Each peptide is displayed based on the mass (in kDa, using a logarithmic scale) and migration time (in minutes). The average relative abundance is represented by the peak intensity. To depict also the lower abundant peptides, the same 107 peptides with abundance magnified 10-fold are shown on the right. Panel A shows the distribution of the 107 peptides before treatment, panel B depicts the distribution after treatment. To ease assessment of changes, the data from patients before and after treatment were superimposed in panel C, peaks were labeled red before EMPA treatment and green after EMPA treatment, the yellow fraction of the peak indicates the overlap in abundance between before and after treatment.

All available samples were analyzed blinded. All data on the individual samples are available in **S1 Table**. To increase statistical power, data from baseline samples collected during clamped euglycemia and hyperglycemia were combined for the analysis, and the same was done for all follow-up samples obtained after empagliflozin treatment for 8 weeks. The investigation of the proteome of these 160 samples enabled identification of 107 peptides that changed significantly between the baseline and post-treatment samples (**S2 Table**). **Fig 1** illustrates the distribution of the 107 peptides before and after treatment with empagliflozin.

To obtain information on the potential relevance of these changes in the context of CKD, the distribution of these peptides in the cohort of patients with CKD and controls that was used to define CKD273 was investigated. This cohort consisted of 379 healthy controls, and 230 patients with CKD of various etiologies (including 50 patients with diabetic nephropathy) [8]. Of the 107 peptides affected by empagliflozin, 79 showed a greater than 25% change in abundance (either increase or decrease) before and after treatment, and between CKD patients and healthy controls. In the next step we compared the directional change (up- or down-regulation) induced by empagliflozin to the change observed in CKD. The underlying hypothesis was that a change in the same direction in CKD and as a result of empagliflozin treatment may indicate a potential negative effect of empagliflozin, (by inducing a similar response than CKD) and a change in the opposite direction may indicate a potential positive effect (a change towards “healthy”). In 60 of the 79 peptides the change after empagliflozin treatment was opposite to the change observed in CKD, indicating a potential beneficial effect. For 19 peptides, the empagliflozin-induced change was similar to the change observed in CKD.

Amino acid sequences were obtained for 46 of the 79 peptides, all listed in **Table 1**. When investigating these 46 sequenced peptides only, the significant changes in peptides derived from clusterin, alpha-1-antitrypsin, keratin type 2, and mucin were in the direction towards “healthy”, when comparing healthy controls and CKD. The results were less consistent when investigating the change in collagen fragments induced by empagliflozin: larger collagen fragments generally appeared to be increased upon treatment, while smaller fragments appear to be decreased, possibly indicating reduction of specific protease activity involved in the processing of collagen.

**Table 1:** Lists the 46 peptides significantly different prior to and after treatment with empagliflozin.

Mass	CE-T	Sequence	Protein Name	Pre EMPA	Post EMPA	EMPA-ind change	BH adj p-value	CKD mean	control mean	CKD-induced
**2320**	**20,8**	**KNGDDGEAGKpGRPGERGppGPQ**	**Collagen alpha-1(I) chain**	**219,08**	**305,54**	**1,39**	**0,0491**	**43,45**	**148,32**	**0,29**
**2328**	**21**	**EDPQGDAAQKTDTSHHDQDHP**	**Short peptide from AAT**	**221,04**	**328,15**	**1,48**	**0,0479**	**20,08**	**123,84**	**0,16**
**1676**	**29,3**	**PGSSGFpGnPGMKGEAGp**	**Collagen alpha-2(V) chain**	**29,56**	**17,38**	**0,59**	**0,0479**	**40,99**	**35,51**	**1,15**
**1694**	**23,5**	**PpGPpGKNGDDGEAGKpG**	**Collagen alpha-1(I) chain**	**107,8**	**164,62**	**1,53**	**0,0479**	**40,84**	**67,16**	**0,61**
**2216**	**33,8**	**IGPpGPAGApGDKGESGPSGPAGPTG**	**Collagen alpha-1(I) chain**	**387,62**	**493,17**	**1,27**	**0,0467**	**226,02**	**548,87**	**0,41**
**2378**	**23,6**	**KNGDDGEAGKpGRpGERGPPGpqG**	**Collagen alpha-1(I) chain**	**15,55**	**4,04**	**0,26**	**0,0450**	**12,39**	**4,11**	**3,02**
**2068**	**20,6**	**EEDDGEVTEDSDEDFIQP**	**E3 ubiquitin-protein ligase TRIM33**	**1254,5**	**1751,18**	**1,4**	**0,0432**	**626,98**	**1694,11**	**0,37**
**3287**	**25,4**	**AGRpGEVGPpGPPGPAGEKGSPGADGPAGAPGTPGPQG**	**Collagen alpha-1(I) chain**	**24,43**	**5,51**	**0,23**	**0,0432**	**4,65**	**16,00**	**0,29**
**2007**	**21,4**	**GSTGPAGqKGDSGLPGPpGSpGP**	**Collagen alpha-1(XI) chain**	**109,18**	**66,7**	**0,61**	**0,0422**	**15,50**	**51,67**	**0,30**
**906,4**	**24,8**	**GPpGPpGPpS**	**Collagen alpha-1(XVIII) chain**	**34,59**	**21,06**	**0,61**	**0,0367**	**37,01**	**19,31**	**1,92**
**1657**	**25**	**GSPGGKGEmGpAGIpGApG**	**Collagen alpha-1(III) chain**	**18,51**	**5,79**	**0,31**	**0,0351**	**9,73**	**7,10**	**1,37**
**3281**	**36**	**GpAGQDGVGGDKGEDGDPGQPGPpGpSGEAGpPGPPG**	**Collagen alpha-1(XI) chain**	**1077,2**	**817,32**	**0,76**	**0,0333**	**1755,73**	**1915,38**	**0,92**
**1401**	**22,6**	**DGESGRpGRpGER**	**Collagen alpha-1(III) chain**	**15,51**	**3,84**	**0,25**	**0,0333**	**19,55**	**20,43**	**0,96**
**1751**	**23,9**	**GpPGpPGKNGDDGEAGKpG**	**Collagen alpha-1(I) chain**	**1215,9**	**1808,18**	**1,49**	**0,0333**	**1022,81**	**1215,80**	**0,84**
**3225**	**22,7**	**EqGHpGSPGFKGIDGMPGTPGLKGDRGSPGmDG**	**Collagen alpha-2(IV) chain**	**27,84**	**11,96**	**0,43**	**0,0317**	**17,76**	**28,65**	**0,62**
**1697**	**29,6**	**pGNDGAKGDAGAPGAPGSqG**	**Collagen alpha-1(I) chain**	**41,69**	**21,5**	**0,52**	**0,0286**	**28,06**	**24,27**	**1,16**
**1517**	**41**	**FDSDPITVTVPVEV**	**Clusterin**	**49,09**	**279,09**	**5,69**	**0,0286**	**110,21**	**210,33**	**0,52**
**3272**	**32,1**	**GEVGPAGSPGSNGApGQRGEPGPQGHAGAQGPPGpPG**	**Collagen alpha-1(III) chain**	**29,08**	**11,04**	**0,38**	**0,0279**	**6,39**	**6,10**	**1,05**
**1585**	**24,6**	**EGSpGHPGQpGPpGPpG**	**Collagen alpha-1(III) chain**	**9,68**	**20,45**	**2,11**	**0,0277**	**6,24**	**14,97**	**0,42**
**1928**	**19,5**	**DGPRGpTGPIGPPGpAGQPGD**	**Collagen alpha-1(III) chain**	**175,34**	**91,85**	**0,52**	**0,0273**	**29,02**	**67,23**	**0,43**
**1142**	**37,3**	**GPpGpPGPPGPpA**	**Collagen alpha-1(VIII) chain**	**492,94**	**696,2**	**1,41**	**0,0233**	**134,19**	**404,17**	**0,33**
**1239**	**21,2**	**GEAGHPGPPGPpGP**	**Collagen alpha-1(V) chain**	**26,03**	**6,73**	**0,26**	**0,0230**	**14,04**	**32,88**	**0,43**
**2529**	**27**	**GETGPAGRpGEVGPpGPpGPAGEKGSpG**	**Collagen alpha-1(I) chain**	**75,5**	**37,54**	**0,5**	**0,0227**	**34,58**	**48,69**	**0,71**
**883,4**	**23,5**	**PpGENGKpG**	**Collagen alpha-1(III) chain**	**81,46**	**56,76**	**0,7**	**0,0224**	**52,08**	**72,76**	**0,72**
**3417**	**32**	**GPpGADGQPGAKGEpGDAGAKGDAGPPGpAGPAGPPGpIG**	**Collagen alpha-1(I) chain**	**1172,9**	**1495,4**	**1,27**	**0,0211**	**692,86**	**1154,42**	**0,60**
**1080**	**27,6**	**DRGEpGPpGPA**	**Collagen alpha-1(I) chain**	**206,04**	**281,42**	**1,37**	**0,0197**	**45,22**	**145,01**	**0,31**
**2487**	**28,2**	**GADGQPGAKGEpGDAGAKGDAGPpGPAGP**	**Collagen alpha-1(I) chain**	**646,01**	**460,79**	**0,71**	**0,0197**	**230,53**	**418,62**	**0,55**
**2047**	**32,8**	**GSNGNpGpPGPSGSpGKDGPpGP**	**Collagen alpha-1(III) chain**	**1282,1**	**827,74**	**0,65**	**0,0192**	**1585,20**	**1925,74**	**0,82**
**3686**	**22,2**	**GRPEAQPPPLSSEHKEPVAGDAVPGPKDGSAPEVRGA**	**Neurosecretory protein VGF**	**1482**	**2353,74**	**1,59**	**0,0169**	**1426,76**	**1378,98**	**1,03**
**1496**	**23,5**	**GPpGPpGPpGPpGPPSA**	**Collagen alpha-1(I) chain**	**60,6**	**29,67**	**0,49**	**0,0169**	**98,77**	**72,92**	**1,35**
**2825**	**24,5**	**ERGEAGIpGVpGAKGEDGKDGSpGEpGANG**	**Collagen alpha-1(III) chain**	**19518**	**22836,6**	**1,17**	**0,0155**	**######**	**16825,06**	**0,92**
**2663**	**23,6**	**NRGERGSEGSPGHpGQpGPPGpPGApGP**	**Collagen alpha-1(III) chain**	**1707,5**	**1298,97**	**0,76**	**0,0148**	**720,20**	**1021,11**	**0,71**
**1262**	**38**	**EAVGDGDGDGDADA**	**ATPase WRNIP1**	**200,94**	**94,99**	**0,47**	**0,0145**	**26,07**	**106,33**	**0,25**
**1484**	**22,6**	**GPpGKNGDDGEAGKpG**	**Collagen alpha-1(I) chain**	**360,21**	**704,15**	**1,95**	**0,0135**	**135,73**	**318,75**	**0,43**
**1792**	**30,8**	**RGApGpDGNnGAQGPpGPQ**	**Collagen alpha-2(I) chain**	**83,11**	**24,34**	**0,29**	**0,0094**	**53,75**	**47,05**	**1,14**
**1977**	**19,3**	**NSESSTVSSGASTATTSESST**	**Mucin-21**	**186,17**	**379,32**	**2,04**	**0,0092**	**60,40**	**89,04**	**0,68**
**2088**	**19,4**	**GPPGSPGEDGpAGEpGPPGpEGq**	**Collagen alpha-1(XV) chain**	**208,07**	**368,2**	**1,77**	**0,0092**	**123,45**	**250,72**	**0,49**
**1532**	**39,3**	**GLpGPpGSNGNpGPpGP**	**Collagen alpha-1(III) chain**	**514,41**	**339,07**	**0,66**	**0,0092**	**258,97**	**322,87**	**0,80**
**1935**	**19,9**	**GSGGSSYGSGGGSYGSGGGGGGGRG**	**"Keratin; type II cytoskeletal 1"**	**553,01**	**917,44**	**1,66**	**0,0092**	**182,62**	**633,17**	**0,29**
**1116**	**36,9**	**PpGPpGpPGPpS**	**Collagen alpha-1(I) chain**	**4**	**30,72**	**7,68**	**0,0090**	**68,83**	**78,96**	**0,87**
**1539**	**29,7**	**PpGEAGKpGEQGVpGD**	**Collagen alpha-1(I) chain**	**236,76**	**168,38**	**0,71**	**0,0082**	**156,03**	**253,95**	**0,61**
**3209**	**22,7**	**PpGESGREGApGAEGSpGRDGSpGAKGDRGETGP**	**Collagen alpha-1(I) chain**	**5616,4**	**4002,63**	**0,71**	**0,0077**	**3257,70**	**6895,61**	**0,47**
**2048**	**19,9**	**KGEDGDPGqpGPPGpSGEAGpP**	**Collagen alpha-1(XI) chain**	**25,37**	**68,37**	**2,69**	**0,0070**	**0,00**	**20,53**	**0,00**
**2679**	**23,6**	**PGMPGADGpPGHPGKEGppGEKGGQGpPG**	**Collagen alpha-1(V) chain**	**2842,7**	**2158,62**	**0,76**	**0,0068**	**2330,40**	**2398,19**	**0,97**
**2695**	**23,5**	**AGSPGSNGApGQRGEpGpQGHAGAqGPPGP**	**Collagen alpha-1(III) chain**	**2652,7**	**2079,6**	**0,78**	**0,0068**	**3134,21**	**2728,53**	**1,15**
**3554**	**31,1**	**GPAGpAGERGEQGPAGSpGFqGLPGPAGPPGEAGKPGEq**	**Collagen alpha-1(I) chain**	**72,83**	**24,19**	**0,33**	**0,0068**	**27,93**	**54,70**	**0,51**

Mass, migration, and sequence of the individual peptides are given as identifiers. The distribution before and after empagliflozin treatment, expressed as relative abundance is given, the induced fold-change, and the p-value after adjustment for multiple testing. In addition, the distribution of these peptides in a cohort of patients with CKD and controls [8], as well as the observed fold-change in this cohort is listed.

**Fig 2:**
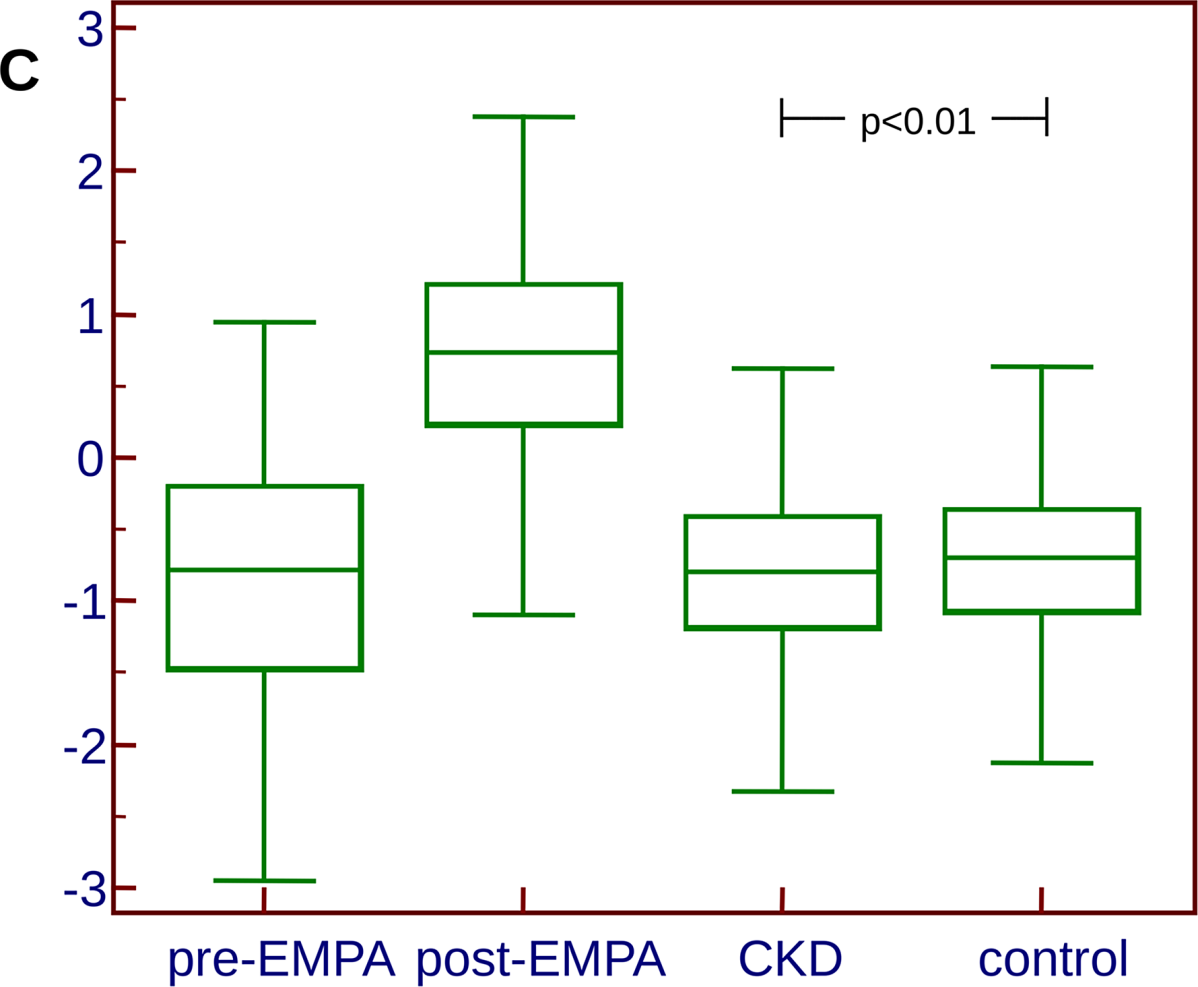
Box-Whisker blots of the scoring representing the proteome changes induced as a result of empagliflozin treatment. The clear separation of the groups prior and after treatment is evident. However, when applying this classifier to a cohort of CKD patients and controls, the controls score slightly, yet significantly (p<0.01) higher (indicative of empagliflozin treatment) than the CKD patients.

To investigate how the changes in these 107 peptides relate to CKD, we combined all 107 peptides into an SVM-based classifier to receive a composite score, using the data obtained from all samples investigated in the context of empagliflozin treatment. The classifier was trained using all 160 samples, and allowed clear differentiation between the two groups (treated and untreated) in the training set. This classifier was subsequently applied onto the 609 samples that were also employed in the discovery of CKD273 in the past [8] to investigate if a significant difference in the scoring in the two groups can be detected, which would indicate a potential impact of empagliflozin treatment on CKD (in both directions, either promoting or suppressing its development). In this independent dataset from patients with CKD of different etiologies and controls, the mean scoring of the CKD cases was -0.835, the mean scoring of the controls was -0.711 (p = 0.0075 for the between-group difference, **Fig 2**). These data further support the initial findings, that the changes induced with empagliflozin are directional pointing away from CKD and towards the healthy controls (of the 107 peptides significantly changing in abundance after empagliflozin treatment, 60 peptides pointing towards healthy, 19 pointing towards CKD, 28 appear unchanged, as outlined above), indicating a potential benefit of empagliflozin.

**Fig 3:**
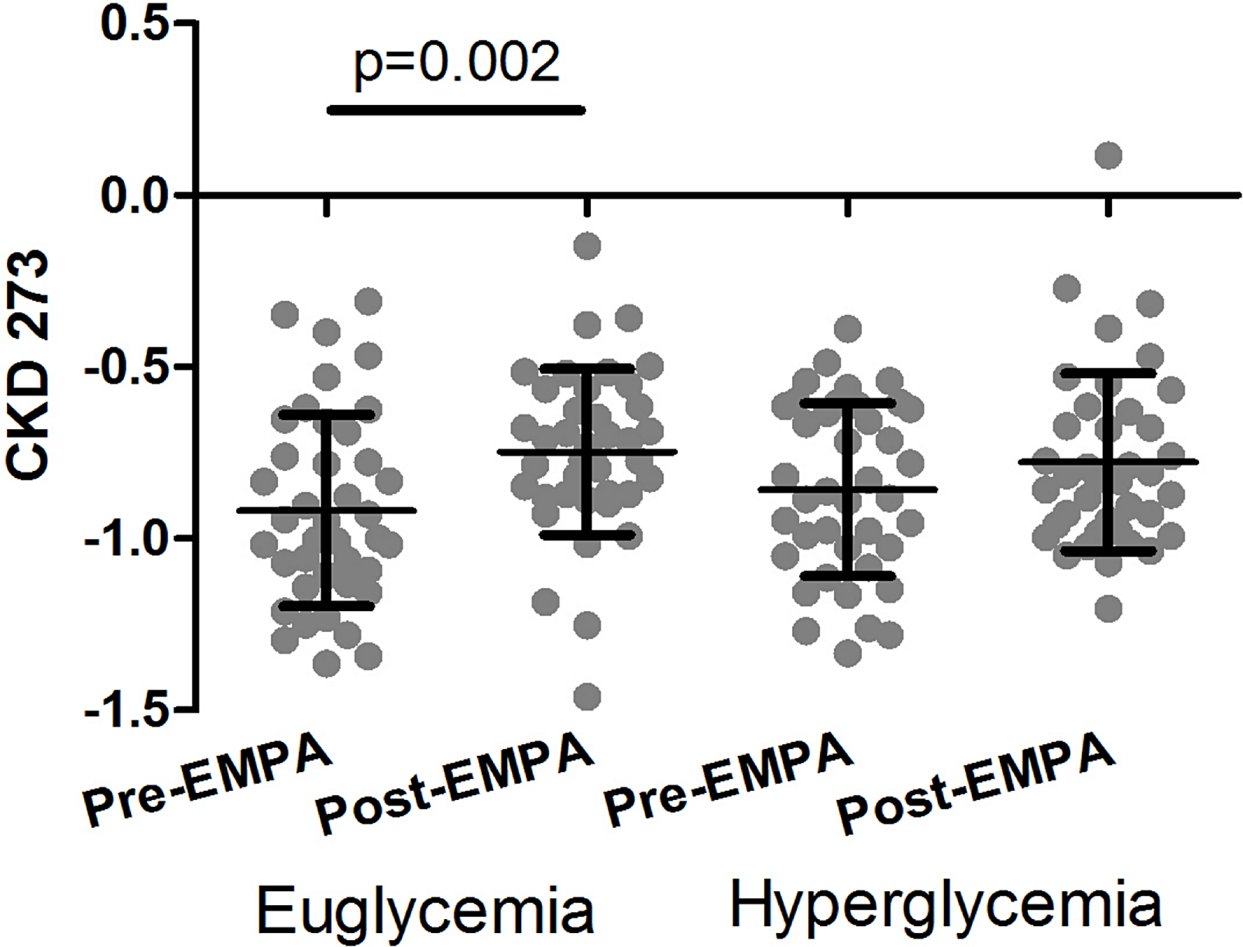
Distribution of the CKD273 scoring in patients before and after treatment with empagliflozin.

To further assess the effect of empagliflozin, we applied CKD273, a high-dimensional classifier based on 273 urinary peptides that were found significantly changed in CKD [8,22], onto the 160 datasets obtained in this study. All baseline samples scored negative, indicating absence of CKD, in line with the clinical findings. As shown in **Fig 3**, levels of CKD273 increased within the negative (CKD absent) range during clamped euglycemia, but not during clamped hyperglycemia. Overall, no consistent impact of the empagliflozin treatment on CKD273 could be observed in this cohort.

## Discussion

In this analysis we investigated the impact of empagliflozin treatment on the urine proteome in type I diabetes patients with preserved kidney function. In several recent studies urinary proteomic changes were reported as a consequence of therapeutic intervention, both based on drugs [13,23], but also on diet or lifestyle [24,25]. Our aim was to determine if a) empagliflozin treatment has an impact on urinary peptides, and b) if such an impact of empagliflozin treatment would be in the direction of CKD (indicating a negative effect) or towards “healthy” (indicating a positive effect). We have in several recent studies identified collagen type I fragments as being decreased in the urine in the initial phase of onset of CKD [9,11]. Though this process was not consistently affected by empagliflozin treatment, among the collagen fragments, larger fragments generally showed changes in the same direction observed in CKD, while the smaller fragments generally showed changes towards healthy. Further, we detected an increase in a specific clusterin peptide after empagliflozin treatment. Peptides derived from clusterin were found decreased in CKD [8], indicating that the observed change presented here represents an improvement with respect to CKD development.

Of special interest is the upregulation of a mucin fragment as a result of empagliflozin treatment. In a recent manuscript we have identified the decrease of mucin fragments in urine as a major component in the CKD-induced changes [26]. The data presented here indicate that empagliflozin treatment may reverse these CKD-induced changes. To assess the impact of the combined changes in urinary peptides observed as a result of empagliflozin treatment, we generated a composite classifier integrating all 107 peptides. When applying this classifier onto an independent cohort of healthy controls and patients with CKD, we detected a significantly higher scoring in the group of healthy individuals, indicating a positive impact of empagliflozin.

Empagliflozin is the first compound that to our knowledge demonstrates a rapid and significant impact on urinary peptides in the context of diabetes and CKD. In our previous investigations, while short term treatment with irbesartan [23] did not show a significant impact on urine peptides, longer-term treatment for 2 years did show an impact. No overlap exists between the changes induced by irbesartan and the changes observed here as a result of empagliflozin, suggesting that empagliflozin has a different, direct impact on urinary peptides, although the mechanisms responsible for these changes are not yet known.

We also investigated the impact of empagliflozin treatment on CKD273, a urinary peptide-based classifier associated with CKD onset and progression [9,11,17]. All patients in the study scored negative for CKD. While this observation was in agreement with the clinical characteristics, we did expect that at least some patients would present borderline or positive scoring for CKD. In these patients, we hypothesized to see an impact of empagliflozin treatment on the classification, a change towards “healthy”. However, all samples scored “healthy” in this primary prevention cohort, and significant improvement of the “healthy” scoring as a result of empagliflozin treatment was not to be expected. The CKD273 score increased within the “CKD absent” range during clamped euglycemia, and we were unable to detect an impact of empagliflozin on CKD273 during clamped hyperglycemic conditions. Since levels of CKD273 were all in the CKD absent range at all studied time points in this cohort, it is difficult to draw any conclusions about prognostic implications in patients with baseline CKD.

This exploratory, *post-hoc* study has limitations: The sample size was small and the treatment period was only 8 weeks, perhaps not sufficiently long to observe significant changes in CKD273. The results may not be generalizable to patients with either type 2 diabetes or patients with evidence of renal disease. Whether SGLT2 inhibition impacts on CKD273 in patients with a background of renal disease is unknown, and will be examined in future work.

In conclusion, empagliflozin has a significant impact on specific urine peptides, and the proteomic changes induced by empagliflozin suggest a potential nephroprotective effect. However, this potential nephroprotective effect needs to be investigated and confirmed in dedicated clinical trials that are designed to assess the impact of empagliflozin in patients with CKD, and investigate how these proteomic changes correlate with clinical outcomes.

## Supporting information

S1 Table In this table the normalized relative abundance of all peptides or proteins identified in this study is listed, for all subjects. Original ID of the samples is given on the top. For all peptides or proteins, internal Peptide ID, mass and migration is given. In addition, sequence, original protein, as well as start and stop amino acid position is given, where applicable.
(XLSX)

S2 Table Listed are the 107 peptides found significantly changes upon empagliflozin treatment. The table lists the mass (in Da) and migration time (CE-T, in minutes), the relative abundance before (Pre EMPA) and after (Post EMPA) treatment, and the fold-change induced by empagliflozin (EMPA-ind change). Further, the average abundance in patients with CKD (CKD mean) and controls (control mean) as well as the fold-change observed in CKD (CKD-induced) is listed. Where applicable, the Sequence, and protein Name and Symbol are given. For all peptides, the unadjusted p-value (for the difference prior and after empagliflozin treatment), the AUC, and the Benjamini-Hochberg (BH) adjusted p-value is listed.
(XLSX)
